# Effect of carotenoid encapsulation on antioxidant activities: A protocol for systematic review

**DOI:** 10.1097/MD.0000000000019772

**Published:** 2020-04-17

**Authors:** Jaluza Luana Carvalho de Queiroz, Isaiane Medeiros, Grasiela Piuvezam, Ana Clara de França Nunes, Camila Carvalho Gomes, Bruna Leal Lima Maciel, Ana Heloneida de Araújo Morais, Thaís Souza Passos

**Affiliations:** a Biochemistry Postgraduate Program, Biosciences Center; b Nutrition Postgraduate Program, Center for Health Sciences; c Public Health Postgraduate Program, Center for Health Sciences; d Department of Nutrition, Center for Health Sciences, Federal University of Rio Grande do Norte, Natal, RN, Brazil.

**Keywords:** antioxidant, carotenoids, oxidative stress, oxygen radical absorbance capacity, reactive oxygen species, systematic review

## Abstract

**Background:**

Carotenoids play essential roles in human health, such as antioxidant activity, and therefore can decrease free radicals oxidation action, preventing numerous diseases. However, these compounds have an unstable nature, turning them susceptible to adverse conditions in food processing and storage. Thereby the search for alternatives that maintain and enhance carotenoid antioxidant function, such as encapsulation, has grown. The objective of this study was to establish a systematic review protocol to evaluate the effect of different encapsulation techniques on the antioxidant action of carotenoids, evaluating which one is the best and safest, and their role in enhancing the antioxidant activity.

**Methods:**

This protocol was guided by the preferred reporting items for protocols for systematic reviews and meta-analyzes. The databases to be searched are PubMed, EMBASE, Scopus, ScienceDirect, and Web of Science. Experimental studies conducted in rats and mice (in vivo) of both sexes and ages, evaluating the use of encapsulated and crude carotenoids will be included in the systematic review. The characteristics of the studies, the experimental model, and the main results will be described, and the risk of bias assessment will be evaluated. Three independent reviewers will proceed with the selection of studies, data extraction, and methodological quality assessment. A narrative synthesis will be made for the included studies. Besides, if sufficient qualitative data is available, a meta-analysis will be conducted. I2 statistics will be used to assess heterogeneity.

**Results:**

This protocol will guide the production of a systematic review that can determine the effect of different encapsulation techniques and encapsulating agents on the antioxidant action of carotenoids. Thus, it will enable the determination of the best encapsulation techniques to promote the preservation and increase of the antioxidant activity, contributing to future research that may reproduce the best carotenoid encapsulation technique in an animal model.

**Conclusion:**

The systematic review to be produced from this protocol will provide support for the construction of research that evaluates the effect of encapsulation on the antioxidant function of carotenoids and its possible application as a nutraceutical, considering that this functionality is directly associated with health promotion.

**Record of systematic review:**

This review was recorded in the International Register of Prospective Systematic Reviews on January 22, 2020 (registration: CRD42020142065). Available at: https://www.crd.york.ac.uk/prospero/display_record.php?ID=CRD42020142065

## 1 Introduction

Carotenoids are molecules responsible for the modulation of numerous metabolic activities, thereby providing health benefits related to antioxidant activity.^[1]^ However, they are not produced by the human body, and must be obtained through food and supplementation, which occur mainly by eating vegetables with yellow, orange, or red coloring.^[2]^

The literature shows that the protective and health-promoting effect of carotenoids is directly associated with the ability to promote the scavenging of free radicals through the transfer of energy from the excited electron from the free radical to the carotenoid.^[3]^ However, despite the benefits related to consumption, the phytochemicals bioactivity is limited by low stability associated with food processing and storage.^[4]^ One way to enhance this effect and to increase the forms of use is the isolation and encapsulation of the carotenoids.^[5,6]^

Encapsulation acts to potentiate the effect of carotenoids, increasing solubility in water, stability to heat, pH, presence of light and oxygen and improving antioxidant capacity. Thus, encapsulation turns carotenoids more stable and efficient.^[7–9]^

In contrast, in vivo studies show that the oral administration of these encapsulated compounds improves bioavailability, mainly, enhancing the antioxidant capacity, which protects cells from oxidative and free radical damage generated in tissues (kidneys and liver).^[10,11]^

However, the selection of encapsulating agents and the techniques used in encapsulations are decisive to promote preservation and improvement of the antioxidant activity of these compounds.^[12,13]^ On the other hand, specificities of the techniques used are scarce, being an obstacle in the analysis of the determination of antioxidant activity.

Based on this and, considering the progression of studies on the application of encapsulation in improving the functionality of carotenoids, it is necessary to evaluate the effect of different encapsulation techniques on the antioxidant action of carotenoids, evaluating which one is the best and safest, and their role in enhancing the antioxidant activity. This knowledge is important, considering that the antioxidant activity is directly related to the application of carotenoids as a health-promoting molecule and the use as a nutraceutical, and that animal studies are seen as an essential step for a possible humans clinical application.

## 2 Methodology

### 2.1 Protocol and registration

This protocol has been prepared according to the guidelines described in Preferred Reporting Items for Systematic Reviews and Meta-Analyses Protocols. A 17-item checklist was used to improve the quality of the systematic review data.^[14]^ The protocol was registered with the International Prospective Register of Systematic Reviews on January 22, 2020 (registration: CRD42020142065). Available at: https://www.crd.york.ac.uk/prospero/display_record.php?ID=CRD42020142065

### 2.2 Eligibility criteria

Peer-reviewed journal articles that meet the eligibility criteria based on population, interventions, control, and study results, Table 1, will be included in the review.

**Table 1:**
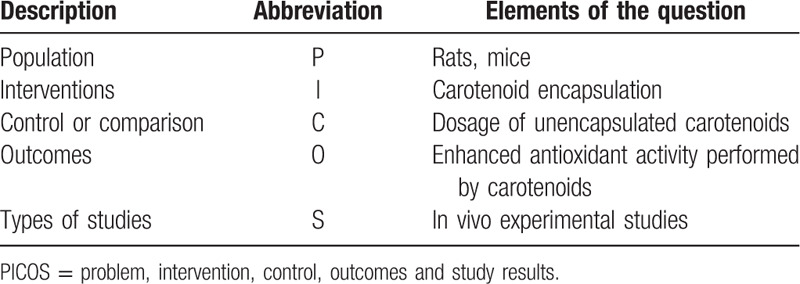
Elements of the research question according to the PICOS strategy.

#### 2.2.1 Inclusion criteria

This review will include original articles resulting from experimental studies conducted in rats and mice (in vivo) without the restriction of water or diet, of both sexes and varied ages, evaluating the use of crude and encapsulated carotenoids.

#### 2.2.2 Exclusion criteria

Review articles, case reports, comments, editorials, letters to the editor, theses, and conference proceedings will be excluded. Studies with other animal models or that do not describe schedules, length of experience, frequency, and doses administered will be excluded. Studies that do not differ from crude and encapsulated carotenoids, and studies without at least one control group will also be excluded.

### 2.3 Information sources and literature search

The electronic search strategy used will include the following databases: PubMed, Science Direct, Scopus, Web of Science, Virtual Health Library, and EMBASE. The search strategy will include the following titles from Medical Subject Headings: “Encapsulation,” “carotenoid,” and “antioxidant” (Table 2).

**Table 2:**
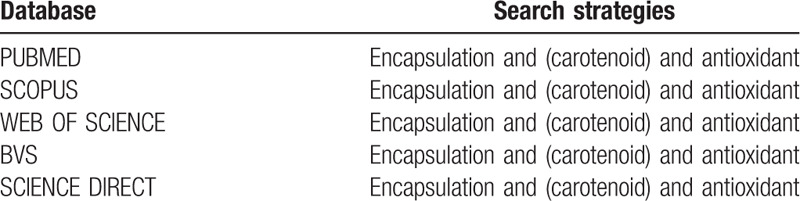
Search strategies for each database.

**Figure 1:**
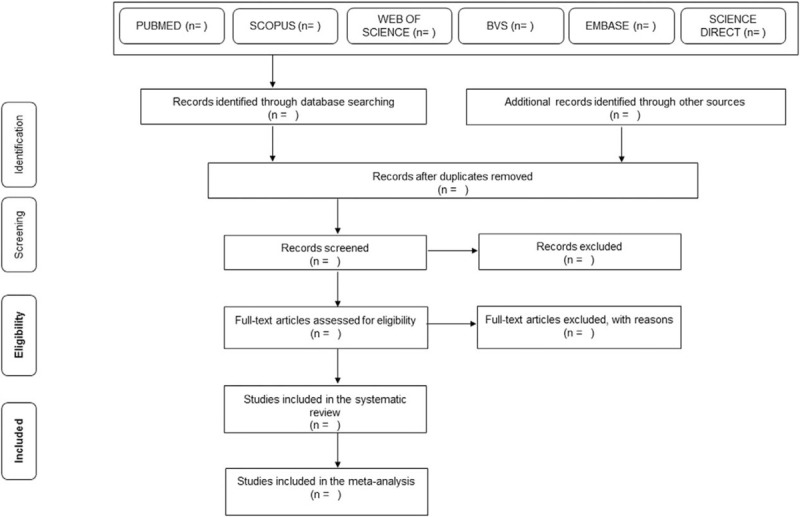
Article selection flow diagram. Adapted from PRISMA-P. [Moher et al, 2015]. PRISMA-P=Preferred Reporting Items for Systematic Reviews and Meta-Analyses Protocols.

The articles will be imported into the Rayyan application (version 0.1.0), and the initial evaluation of the studies will be done using the title and abstract, and the research strategies informed in Table 2, following the inclusion and exclusion criteria, by three independent researchers (JLCQ, CCG, and IM). If an abstract is not provided using the search strategy above, the full text of the manuscript will be reviewed and evaluated. If the three independent researchers disagree with a specific study, a third researcher (ACFN) will decide whether or not to include the research. All researchers will review the full text of all studies considered eligible for inclusion. The study selection was summarized in the Preferred Reporting Items for Systematic Reviews and Meta-Analyses flow diagram (Fig. 1).

References to the included manuscripts will also be reviewed to identify possible missed attempts (manual search).

### 2.4 Data extraction

Data extraction will be performed independently and standardized by 3 researchers (JLCQ, CCG, and IM), using a spreadsheet prepared in the Microsoft Excel Program. The following information will be inserted in the spreadsheet: author, year, type of study, number and type of control groups, time of the experiment, statistical measures used, results, and other information that may be necessary. For any relevant data missing from the manuscripts, contact with the study authors will be attempted. If the required information is not obtained, the data will be excluded from the analysis and covered in the discussion section.

### 2.5 Risk of bias and quality assessment

Three reviewers will assess the risk of bias in the selected studies, and the differences, if any, will be resolved by consulting a fourth reviewer. The Systematic Review Center for Laboratory Animal Experimentation will be used to evaluate the risk of bias.^[15]^ The reviewers will be previously trained and calibrated to ensure uniformity in the evaluation of the criteria.

### 2.6 Data analysis and synthesis

Data will be presented in summary tables and in narrative form to describe the characteristics of the studies. They will be structured around the type of carotenoid, the administered and tested dose, as well as the encapsulation technique, the antioxidant activity evaluation techniques, and the result obtained concerning the antioxidant activity. At the end of the analysis of the data extracted from the included studies (at least 2), if clinical, methodological and statistical homogeneity are found, a meta-analysis will be performed. This will be carried out using the Rev Man Analyses statistical package in Review Manager v. 5.3.

Because of the exploratory nature of animal studies, the random-effects model will be used to account for anticipated heterogeneity. For dichotomous outcomes, we will derive the OR and 95% CI for each study. The heterogeneity between the trial results will be evaluated using a standard *I*^2^ test with a significance level of *P* .1, and the *I*^2^ statistic, which is a quantitative measure of inconsistency across studies, with a value of 0% indicating no observed heterogeneity, and values of 50% showing substantial levels are present. If there is heterogeneity (*I*^2^ 75%), a random-effects model will be used to combine the trials to calculate the relative risk (RR) and 95% CI using the DerSimonian-Laird algorithm in meta for the package, a meta-analysis package for R.

Other study characteristics and results will be summarized narratively if a meta-analysis cannot be performed for all or some of the included studies. If possible, funnel plots will also be used to assess the presence of potential reporting biases, and a linear regression approach will be used to evaluate funnel plot asymmetry.

## 3 Discussion

The literature shows that most of the studies with encapsulated carotenoids use this molecule extracted from the particles to test the antioxidant capacity. Therefore, it isn’t effortless to evaluate the functionality of these compounds after encapsulation.^[12,13]^ Based on this, the systematic review that will be conducted from this protocol will have as the primary purpose to present studies that show the effect of encapsulation on the antioxidant activity of carotenoids.

Encapsulation consists of trapping a molecule (active or core) within an encapsulating agent. This technique aims to improve the functionality of the core, and to provide better stability, increasing the action through controlled release, facilitating handling and protecting the bioactives from adverse conditions such as processing, environmental, and gastrointestinal conditions.^[16,17]^

In this perspective of improving the functionality of the active, one of the main points is the particle size, which is subdivided into microparticles (sizes between 1 to 100 μm) and nanoparticles (sizes between 1 to 100 nm). Nanoparticles due to their smaller size have a series of advantages such as release in specific organs or targets and, therefore, better intracellular uptake and passage through smaller blood vessels, such as capillaries.^[18,19]^

To obtain a better application and, consequently, better results in vivo, such as better absorption, as well as utilization in food and industry, encapsulation techniques and encapsulating agents must be taken into account.^[20]^

Due to the great diversity of molecules, there is no way to point out a technique or universal encapsulating agent, so the choice should consider the bioactive that will be encapsulated. To have better efficiency in the process and a satisfactory result, factors such as the use of non-toxic ingredients, satisfactory cost-benefit, resistance to degradation in the gastrointestinal tract (by hydrolysis or oxidation) must be assessed. Therefore, it is essential that the encapsulating agent, as well as the encapsulation technique, promotes the preservation and stability of the active agent.^[21,22]^

Since the antioxidant capacity of carotenoids is directly related to their health-promoting role, finding ways to preserve and enhance this functionality is crucial for public health.^[23]^ In this perspective, in vivo studies of encapsulation, characterization, and antioxidant activity are of fundamental importance, as they show whether these encapsulated pigments resist to gastrointestinal conditions, if the antioxidant activity is preserved, and if the release is controlled. Therefore, increasing the efficacy of carotenoids and their bioavailability and bioactivity may lead to an improvement in inflammatory markers, oxidative stress and hepatoxicity. If so, effective encapsulating carotenoids may benefit their use as nutraceuticals.^[24,11]^

Thus, it is necessary to develop a systematic review that identifies the potential of these studies, aiming to guide studies about the effects of carotenoids encapsulation on their antioxidant activities. The compilation of the main findings of the scientific literature will direct current and future researches who work in this area of knowledge.

## Author contributions

JLCQ worked on the review project and wrote the review protocol. IM, JLCQ, ACFN and CCG elaborated the search strategy in the literature. GP and AHAM provided the methodological knowledge. GP, BLLM, TSP and AHAM critically reviewed the methodology and the manuscript. All authors read and approved the final manuscript.

Ana Heloneida de Araújo morais orcid: 0000-0002-6460-911X.
